# Study of Respiratory Disorders in Endoscopically Negative and Positive Gastroesophageal Reflux Disease

**DOI:** 10.4103/1319-3767.61233

**Published:** 2010-04

**Authors:** Maha M. Maher, Amr A. Darwish

**Affiliations:** Pulmonary Unit, Department of Internal Medicine, Mansoura University, Egypt; 1 Menoufyia University Mansoura, Egypt

**Keywords:** Gastroesophageal reflux disease, NERD, respiratory symptoms

## Abstract

**Background/Aim:**

The relation between respiratory disorders and reflux symptoms has been debated since the beginning of the last century and the interest in this question has increased during the last few decades. This study aims to investigate the relation between specified respiratory disorders and reflux symptoms and examine the correlations between respiratory disorders and endoscopic findings in patients with gastroesophageal reflux disease.

**Patients and Methods:**

This study included 515 patients evaluated for gastroesophageal reflux disease (GERD) by patient self-report symptom questionnaire; modified four grade Likert scale and endoscopic assessment using endoscopic Los Angeles Classification. All participants were asked about various respiratory symptoms experienced during the past six months and exposed to measuring body mass index (BMI), medical history, pulmonary physical examination, chest X-ray, respiratory function tests and available sleep studies.

**Results:**

A total number of 515 patients were categorized according to endoscopic findings into two groups; (group1) subjects with normal endoscopic studies (NERD) 118 (22.9%) patients and (group2) subjects with abnormal endoscopic studies (ERD) 397 (77.1%). The proportion of females was significantly higher in ERD group (80.1%) as compared with NERD group (62.7%) (*P*<0.02). Duration of reflux symptoms found to be significantly prolonged in ERD group (*P*<0.03). The cases of ERD group were more likely to be overweight (BMI > 25) *P*<0.02. History of pulmonary symptoms preceding GERD symptoms was found in 15% of patients. There were 294 patients (57.1%) with different pulmonary manifestations. These manifestations were significantly higher among female group (*P*<0.01) and among obese, above 40 years old (*P*<0.001, 0.05 respectively). Among all patients with respiratory manifestations the commonest disorders diagnosed were chronic pharyngitis (50.3%), chronic bronchitis (15.8%), bronchial asthma (12.6%) and recurrent pneumonia (3.3%). Obstructive sleep apnea and recurrent hemoptysis were present in 2.7% and 1.5% of the studied patients respectively. There were three cases of chronic lung abscess. There was a significant difference between ERD and NERD groups in their relations to respiratory disorders (*P*<0.001). There were statistically significant differences in FEV1, FVC and FEV1/FVC (*P*<0.02, *P*<0.05 and *P*<0.05) respectively in ERD group as compared with NERD group.

**Conclusion:**

The study confirms the strong link between gastroesophageal reflux symptoms and various respiratory disorders. Endoscopy of the upper digestive tract remains an important exam in the evaluation of GERD. Respiratory symptoms are more prevalent among erosive esophagitis patients with a positive correlation with degree of severity. There is direct relationship between the severity of airways obstruction as detected by FEV_1_ and FEV1/FVC and GER symptoms.

Gastroesophageal reflux disease (GERD), defined as the presence of symptoms or lesions that can be attributed to the reflux of gastric contents into the esophagus, is one of the most common disorders affecting the gastrointestinal tract. Patients with GERD commonly have symptoms, with approximately 20% experiencing heartburn, acid reflux or both at least once a week and approximately 40% reporting that such symptoms occur at least one a month. If extra- esophageal manifestations are taken into consideration, it is believed that the real prevalence of pathological reflux might be underestimated.[1]

Unlike the distal esophagus, the airways are not protected by antireflux clearance mechanisms and intrinsic mucosal properties. It is therefore conceivable that even a single reflux episode extending beyond the esophagus may be sufficient to cause pharyngeal, laryngeal, and respiratory symptoms and signs. A second mechanism responsible for GERD is activation of reflexes involving the airways by reflux of gastric contents into the esophagus.[2]

The endoscopic esophageal changes caused by reflux disease are not only helpful diagnostically, but also identify patients exposed to a significant risk of disease chronicity.[3–6] Further, the severity of esophagitis gives useful guidance as to the likelihood of success of a particular treatment.[7]

Therefore, the aim of this study was screening and investigating the relation between specified respiratory disorders and reflux symptoms, and to examine the correlations between respiratory disorders and endoscopic findings in patients with gastroesophageal reflux disease in major tertiary hospital in the eastern region of Saudia Arabia.

## PATIENTS AND METHODS

The study included 515 patients presenting to the gastroenterology clinics of King Fahad Hospital Hofuf (a 500-bed, major tertiary-care center in the eastern region of Saudi Arabia) from November 2004 to November 2008.

Patient inclusion criteria were chronic heartburn as the presenting symptom and suspected GERD not treated at the time of the evaluation. Exclusion criteria were smoking, pulmonary malignancies, pregnancy, or laryngeal stenosis. There were 123 (23.9%) male patients and 392 (76.1%) female patients, with an age range of 24-58 years, and mean age of 41.6±7.4 years.

### Modified four-grade Likert scale

Patient self-report symptom questionnaire was used for assessment of GERD. Modified Likert scale[8] with defined individual response options and structured patient self- report, rather than physician assessment, was considered to be the best approach.[9]

### Respiratory symptoms

The participants were asked about various respiratory symptoms experienced during the past six months: (1) Whether daily cough was experienced, and if so, the duration and whether the cough was productive. (2) breathlessness, with three alternative answers: no symptoms, minor degree, or major degree; (3) attacks of heavy breathing or wheezing during the past 6 months; (4) occurrence of asthma; and (5) use of asthma medication, hemoptysis and hoarseness of voice

### Pulmonary function tests

Lung function studies were performed for all the studied cases (515) before doing upper GIT endoscopies using a precalibrated Spirolab II with black/white LCD display spirometer; product from MIR (Medical International Research) Roma, Italy.

All of the following respiratory functions were considered and recorded; forced expiratory volume in the first second (FEV1), forced vital capacity (FVC), FEV1/FVC, peak expiratory flow (PEF) and FEF 25-75 (forced expiratory flow at 25% and 75% of the vital capacity). All of these respiratory functions are reported as percent predicted values.

Furthermore, spirometry results had to be characteristic: forced expiratory volume in one second/forced vital capacity ratio (FEV1/FVC) <70% of predicted, indicating airflow obstruction. Spirometric measurements were performed three consecutive times and the highest value was recorded. These tests were performed in accordance with the Pulmonary Function Test Guidelines established by the European Thoracic Society.[10]

### Endoscopic assessment

Upper GIT endoscopies using Pentax EG – 2940 were done for all cases (515 patients). Based on endoscopic Los Angeles classification[11] of esophagitis, GERD was diagnosed endoscopically in 397 patients (77.1%) from which there were 79 males (19.9%) and 318 females (80.1%).

All patients were exposed to all of the following: medical history, measuring body mass index (BMI), pulmonary physical examination, chest X-rays, respiratory function tests and sleep studies for the suspected cases of sleep apnea. The patients also reported on the use of theophylline, corticosteroids per os, and acid-suppressive drugs such as proton-pump inhibitors and histamine-2-receptor antagonists. Subjects included in this study (515 patients) were categorized into two groups according to endoscopic findings as: (group 1) subjects with heartburn and normal endoscopic studies (NERD) 118 (22.9%) patients and (group 2) subjects with heartburn and abnormal endoscopic studies (ERD) 397 (77.1%), the demographics of the study population are shown in Table 1.

**Table 1:** Demographics of study population subjects with normal endoscopic studies vs. abnormal endoscopic studies

**Characteristics**	**Subjects with normal endoscopic studies (NERD) (n=118)**	**Subjects with abnormal endoscopic studies (ERD) (n=397)**	***P* value**
Male/female	44/74	79/318	0.02
Mean age (range) (months)	33-56 (43.4)	24-59 (40.3)	0.84
Mean symptom duration (range) (months)	17-39 (23)	32-71 (48)	0.03
BMI, kg/m^2^			
<25	8 (6.8)	33 (8.3)	0.43
25-<30	53 (44.9)	171 (43.1)	0.91
30-<35	55 (46.1)	159 (40.1)	0.36
≥35	2 (1.7)	34 (8.6)	0.02
Diabetes mellitus			
Diabetic	81 (68.6)	243 (61.2)	0.45
Non-diabetic	37 (31.4)	154 (38.7)	0.76
Visit physician for reflux symptoms	9-12 (7)	16-22 (18)	0.13
Visit physician for respiratory symptoms	0-2 (0.3)	3-10 (6)	0.35
Use of PPI	22-34 (29)	59-98 (71)	0.63

BMI: Body mass index; PPI: Proton pump inhibitor. Figures in parenthesis are in percentage.

### Statistical analyses

Statistical analysis was performed using SPSS version 10.1. Continuous variables were described as mean±SD. Group comparisons were made using the Student's *t* test. A *P* value <0.05 was considered statistically significant. Correlation between degree of GERD and pulmonary manifestations was assessed by X2 analysis and Mann-Whitney U tests.

## RESULTS

All the 515 patients answered the questions regarding reflux symptoms using modified Likert scale; 73 had mild reflux symptoms, 305 with moderate grade and 137 patients had severe reflux symptoms, females constituted (76.1%) of patients. All patients underwent upper endoscopy and were assessed based on Los Angeles Classification of esophagitis.

According to the endoscopic findings, patients were categorized into (group 1) cases with heartburn and normal endoscopic studies (NERD) 118 (22.9%) patients and (group 2) cases with heartburn and abnormal endoscopic studies (ERD) 397 (77.1%). Table 1 outlines some demographic characteristics of the study participants. The mean ages of NERD and ERD were 43.4 years and 40.3 years, respectively. The proportion of females was significantly higher in ERD groups (80.1%) than in the NERD group (62.7%; *P*<0.02). The duration of reflux symptoms was found to be significantly prolonged in ERD group (*P*<0.03). ERD group was more likely to be overweight (BMI>25; *P*<0.02) otherwise no statistically significant difference was noticed between the two groups, with regard to history of diabetes and use of proton pump inhibitor.

### Relations between respiratory disorders and reflux symptoms

**Figure 1:**
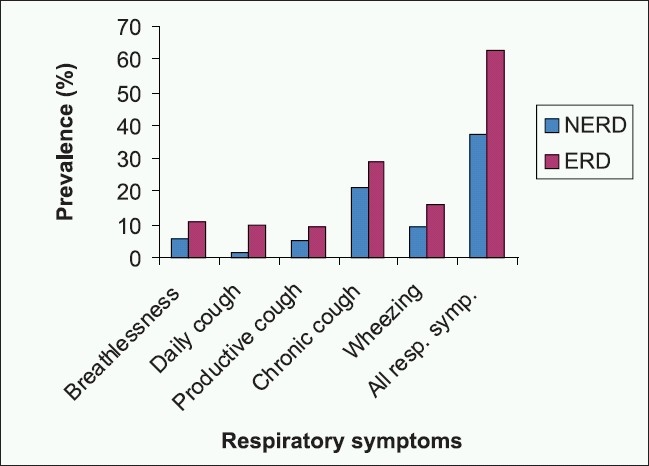
Prevalence of respiratory symptoms in NERD vs ERD patients

Seventy seven patients (15%) (4.9% male and 10.1% female) reported history of pulmonary symptoms that precede GERD symptoms. There were 294 patients (57.1%) with different pulmonary manifestations. These manifestations were significantly higher among female group (*P*<0.01) and significantly higher among obese, above 40 years old (*P*<0.001,0.05 respectively). Prevalence of different respiratory symptoms among NERD and ERD groups is shown in Figure 1.

Strong statistically significant associations between a number of respiratory symptoms and grade of reflux symptoms were found in both groups NERD and ERD [Table 2]. The occurrence of all the respiratory symptoms was about two times more common among ERD patients than NERD patients, especially those complaining of daily cough. As regards disease duration, patients with wheezy breathing, daily cough, daily productive cough, or chronic cough showed a statistically significantly two-fold to three-fold increase in the risk of reflux symptoms.

**Table 2:** Correlation between endoscopic grading and respiratory disorders

**Variables**	**ERD**					**NERD**
		
	**Grade A**	**Grade B**	**Grade C**	**Grade D**	**Total**	
Chronic pharyngitis	32 (21.8)	37 (25.1)	16 (10.9)	9 (6.1)	94 (63.9)*	53 (36.1)
Chronic bronchitis	7 (15.2)	6 (13)	12 (26.1)	15 (32.6)**	40 (87)*	6 (13)
Bronchial asthma	7 (18.9)	8 (21.6)	8 (21.6)	9 (24.3)*	32 (86.5)*	5 (13.5)
Recurrent pneumonia	3 (30)	1(10)	2 (20)	0 (0)	6 (60)	4 (40)
Obstructive sleep apnea	2 (25)	0 (0)	1 (12.5)	0 (0)	3 (37.5)	5 (62.5)
Chronic lung abscess	1(25)	2 (50)	0 (0)	0 (0)	3 (75)	1 (25)

Data are presented as No. (%)

* *P*<0.001

** *P*<0.003

Among all patients with respiratory manifestations, the commonest disorders diagnosed were chronic pharyngitis (50.3%), chronic bronchitis (15.8%), bronchial asthma (12.6%) and recurrent pneumonia (3.3%). Also, obstructive sleep apnea was diagnosed in 2.7%; 1.5% of the patients presented with recurrent hemoptysis. Further, there were three cases of chronic lung abscess.

There was a significant difference between ERD and NERD groups in their relations to respiratory disorders (*P*<0.001). Significant positive correlation between endoscopic grading according to Los Angeles Classification and respiratory symptoms; grade C and D significantly correlated with symptoms of asthma and chronic bronchitis (*P*<0.001, 0.003 respectively) Table 2.

### Pulmonary function tests in NERD and ERD groups

Results of pulmonary function studies were measured in both groups (NERD group and ERD group). There were statistically significant differences in FEV1, FVC and FEV1/ FVC (*P*<0.02, *P*<0.05 and *P*<0.05) respectively in ERD group as compared with NERD group, while there were no significant differences between both groups in measuring PEF and FEF 25-27% [Table 3].

**Table 3:** Pulmonary function tests in NERD and ERD groups

**Respiratory function tests**	**NERD (n=118)**	**ERD (n=397)**	***P* value**
FEV1	92.5±32.3	78.4±25.4	0.02
FVC	90.4±35.0	77.3±±30.1	0.05
FEV1/FVC	79.1±24.6	68.3±17.5	0.05
PEF	81.8±22.4	77.5±29.8	0.43
FEF 25-75%	85.8±31.1	79.3±26.7	0.15

Values are expressed as (Mean±SD)

In the ERD group, large airway narrowing is more commonly seen than small airway obstruction, which demonstrated more frequent bronchitis and bronchial asthma in ERD group as compared to NERD group.

## DISCUSSION

Gastro-esophageal reflux disease (GERD) is a common disorder caused by the reflux of gastric contents into the esophagus. According to a recent global definition,[12] GERD can cause esophageal and extra-esophageal syndromes, which can co-exist, or not, in the same individual. Respiratory manifestations of GERD represent one of the most prevalent and challenging of these extra-oesophageal syndromes. However, the relationship between reflux and respiratory symptoms is frequently difficult to establish with a high degree of certainty.

GERD should be defined by the presence of reflux oesophagitis (Los Angeles grades A–D) and/or when it causes reflux symptoms that are sufficient to impair quality of life and/or when it is associated with a risk of long term complications.[13] The subjects of the present study were selected on the basis of their chronic heartburn and not respiratory pathology. In the measurement of symptom severity, self-assessed by the patient, we used Likert scale which is considered to be an optimal objective end point.[13] “Likert scale” is commonly used to describe symptom scales such as “none, mild, moderate, severe”. Also, all patients underwent upper endoscopy and were categorized based on Los Angeles classification.

Recent decades have witnessed a dramatic revision of the GERD landscape including manifestations of the disorder that could be seen with the naked eye through an endoscope (erosive reflux disease, ERD) or not seen (non-erosive reflux disease, NERD).[14] NERD is common and may comprise more than 60% of all chronic heartburn sufferers in the community.[15,16]

NERD constitutes (22.9%) of our study population. This is in complete disagreement with other studies, which showed a prevalence of 75% and 80% in two separate studies.[17,18] This may be due to difference in dietary habits. In our study, we depended only on endoscopic findings without studying pH difference. We studied prevalence and relation of respiratory disorders in relation to both ERD and NERD.

Epidemiologic studies show a moderate association between GERD and a range of pulmonary symptoms. A cross- sectional study of heartburn prevalence in 2,200 participants showed that incidence of pulmonary symptoms was slightly elevated among those with frequent GERD compared to those without GERD.[19]

Among all patients studied, 294 patients (57.1%) had different pulmonary manifestations. These manifestations were significantly higher among female group (*P*<0.01) and significantly higher among obese, above 40 years old (*P*<0.001, 0.05 respectively). This prevalence is much higher than what was reported in a previous study, which showed that chronic respiratory symptoms or diseases were present in only 18% of patients with GERD.[20]

In spite of the higher prevalence found in obese patients, this association was independent of BMI, which is consistent with a previous report.[21] Confounding could never be completely ruled out. Obesity, for instance, is a well-known risk factor for reflux that has recently been recognized as a risk factor for asthma as well.[22,23] To reduce the risk of confounding, we adjusted statistically for all plausible confounding variables, including obesity represented by BMI.

Asthma is a highly prevalent disease whose incidence has increased in the last decades, affecting 5% to 10% of the global population.[24] There is mounting epidemiological evidence of an association between GERD and asthma, as well as of a strong correlation between reflux episodes and respiratory symptoms. This association has been intensively studied; patients with oesophagitis are more likely to have asthma than patients without esophagitis.[25–27]

Our results showed a statistically significant difference of prevalence of all respiratory symptoms in ERD as compared with NERD groups (63% vs 37.2%, respectively). Moreover, we found significant positive correlation between asthma and chronic bronchitis and endoscopic grading grade C and D (*P*<0.001, *P*<0.003 respectively). Microaspiration of gastric acid and increases in airway hyper-responsiveness due to esophageal acid are considered potential triggers for asthma.[28] A number of reviews reported beneficial results of medical and surgical antireflux therapy on asthma outcome.[29,30]

GERD is currently considered the third leading cause of chronic cough, affecting an estimated 20% of patients.[31,32] Our results showed that 27.2% of our study population complain of a chronic cough, with the frequency being higher for ERD compared to GERD (29% vs. 21.2%, respectively).

The cause-and-effect relationship between GERD and chronic cough is controversial for some authors, as is GERD- induced asthma. However, the principal evidence that GERD is the cause of chronic cough is based on the resolution of the symptoms after an efficacious antireflux treatment.[33]

Accordingly, some authors,[34] contend that neither endoscopy of the upper digestive tract, the gold standard for the diagnosis of GERD complications, nor contrast enhanced radiological examination of the esophagus, stomach and duodenum, are capable of establishing a causal relationship between GERD and chronic cough.[35] However, our study reflects a significant prevalence of chronic cough in patients with erosive esophagitis.

To our knowledge, pulmonary function tests were studied only in patients with chronic respiratory diseases complaining from reflux symptoms. In our study we did pulmonary function tests to all patients provisionally diagnosed as GERD and we studied the relation between pulmonary function tests and the ERD and GERD subgroups.

In this study, there were statistically significant differences in FEV1, FVC and FEV1/FVC (*P*<0.05 and *P*<0.05*,* respectively) in ERD as compared with NERD. On the other hand, there were non significant differences between both groups in measuring PEF and FEF 25-27%. Therefore, these results illustrated that the large airway obstruction is more involved in ERD group than small airway narrowing, which is supported by more frequent bronchitis and bronchial asthma in ERD group as compared to NERD group.

Vraney and Pokorny[36] measured pulmonary functions in patients with gastroesophageal reflux. Results of pulmonary function studies were grouped according to smoking history and demonstrated reflux. The difference noted between the smoker and nonsmoker groups was slightly greater than that between the reflux and non-reflux groups, one of the strength points in this study is exclusion of smokers.

Atalay *et al.*[37] evaluated respiratory function tests (RFTs) in acid reflux positive and -negative patients diagnosed with 24 h pH monitorization. RFTs of reflux-positive patients were not significantly different from those of reflux-negative patients. They concluded that both lung disease and GER have a high prevalence worldwide, and these conditions are frequently coexistent.

The difference between the two previous studies and our study is, firstly, the presence of a higher percentage of our patients with pulmonary symptoms, and secondly, our dependence on mainly endoscopic parameters for diagnosing GERD, and not on pH monitoring. These are mainly the causes for significant respiratory function values in our study as compared with other studies.

The strength of this study lies firstly, in sub-grouping the patients into ERD and GERD, and finding a correlation between respiratory symptoms and these groups, and secondly, in evaluating the respiratory functions in non- smokers.

In conclusion, this prospective study has revealed a strong link between gastroesophageal reflux symptoms and various respiratory disorders. Endoscopy of the upper digestive tract remains an important element in the evaluation of GERD. Respiratory symptoms are more prevalent among erosive esophagitis patients, with a positive correlation with the degree of severity. There is a direct relationship between the severity of airways obstruction as detected by FEV_1_ and FEV1/FVC with GER symptoms.
